# Supplementary material for: Dynamics and significance of the antibody response to SARS-CoV-2 infection
Source: medRxiv. 2020 Jul 20:2020.07.18.20155374. Preprint. [Version 1] doi: 10.1101/2020.07.18.20155374 (PMC7386524; doi:10.1101/2020.07.18.20155374)

medRxiv preprint doi: https://doi.org/10.1101/2020.07.18.20155374.this version posted July 20, 2020. The copyright holder for this preprint (which was not certified by peer review) is the author/funder, who has granted medRxiv a license to display the preprint in perpetuity.
It is made available under a CC-BY 4.0 International license .

Supplementary Materials

medRxiv preprint doi: https://doi.org/10.1101/2020.07.18.20155374.this version posted July 20, 2020. The copyright holder for this preprint (which was not certified by peer review) is the author/funder, who has granted medRxiv a license to display the preprint in perpetuity.
It is made available under a CC-BY 4.0 International license .

Method S1. Additional details on laboratory assays

Enzyme-linked immunosorbent assay (ELISA). The RBDs were expressed in Expi293F suspension cells with a C-terminal SBP-His8X tag, and purified using affinity chromatography and then size exclusion chromatography prior to removal of the His tag as described previously[1]. Briefly, 384 well Nunc MaxiSorp plates (Invitrogen, Carlsbad, CA) were coated by adding 50 µL of RBD in carbonate buffer (1 µg/mL) and incubating for 1 hour at room temperature (RT). Plates were then blocked for 30 minutes at RT with 5% non-fat milk in tris-buffered saline (TBS). Diluted samples (1:100 in TBS with 5% milk, 0.5% Tween) were added to the plate (25 µL/well) and incubated for 1 hour at 37°C with shaking. Serial 4-fold dilutions to 1:6400 were also included for individuals with high titers. Goat anti-human IgA, IgG, and IgM-horseradish peroxidase conjugated secondary antibodies diluted (Jackson ImmunoResearch, West Grove, PA) at 1:10000 (IgG, IgM) or 1:5000 (IgA) in 5% milk in TBST were then added to plates (25 µL/well) and incubated at RT with shaking for 30 minutes. Bound secondaries were detected using 1-step Ultra TMB (tetramethylbenzidine; ThermoScientific, Waltham, MA, 25 µL/well). Plates were incubated at RT for 5 minutes in the dark before addition of 2 N sulfuric acid stop solution (25 µL/well). The optical density (OD) was read at 450 nm and 570 nm on a plate reader. OD values were adjusted by subtracting the 570 nm OD from the 450 nm OD. We used a standard curve of the anti-SARS-CoV-2 monoclonal, CR3022 [2] ,to calculate the concentration of anti-RBD IgG, IgA, and IgM expressed in µg/mL.

Dried blood spots (DBS). Single donor, sero-negative whole blood, collected in sodium heparin tubes (Becton, Dickinson, NJ), was spiked with heat-inactivated plasma from SARS-CoV-2 patients. Forty microliter (40 µL) droplets were spotted in replicate onto Whatman 903 Protein Saver cards (GE Healthcare, Cardiff, UK). Cards were left to dry overnight at ambient room temperature. The following day, two 6-mm^2 punches were removed from the DBS card using a manual hole-punch and eluted overnight at 4°C with gentle agitation in 133 µL PBS-0.05% Tween 20 pH 7.4 (Sigma-Aldrich, St. Louis, MO).

medRxiv preprint doi: https://doi.org/10.1101/2020.07.18.20155374.this version posted July 20, 2020. The copyright holder for this preprint (which was not certified by peer review) is the author/funder, who has granted medRxiv a license to display the preprint in perpetuity.
It is made available under a CC-BY 4.0 International license .

References

1. Norman M, Gilboa T, Ogata AF, et al. Ultra-Sensitive High-Resolution Profiling of Anti-SARS-CoV-2 Antibodies for Detecting Early Seroconversion in COVID-19 Patients. medRxiv 2020.

2. Wang C, Li W, Drabek D, et al. Publisher Correction: A human monoclonal antibody blocking SARS-CoV-2 infection. Nat Commun 2020;11:2511.

medRxiv preprint doi: https://doi.org/10.1101/2020.07.18.20155374.this version posted July 20, 2020. The copyright holder for this preprint (which was not certified by peer review) is the author/funder, who has granted medRxiv a license to display the preprint in perpetuity.
It is made available under a CC-BY 4.0 International license .

Figure S1: Number of PCR positive cases with a sample taken during each week since symptom onset.

The date of symptom onset could not be determined for three individuals and the severity index was missing for one individual

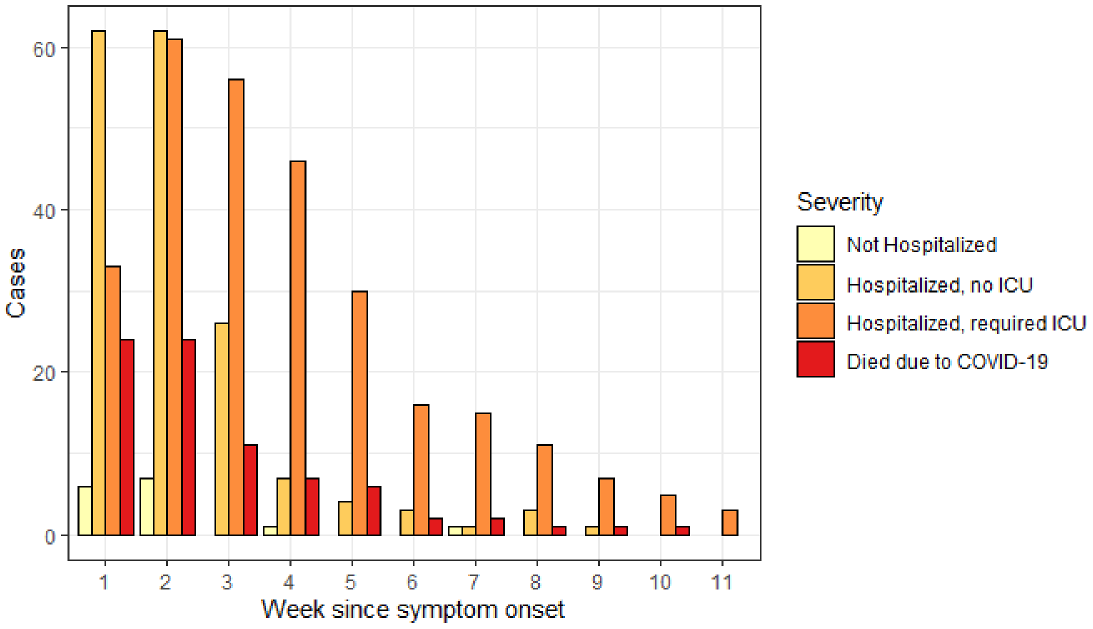

medRxiv preprint doi: https://doi.org/10.1101/2020.07.18.20155374.this version posted July 20, 2020. The copyright holder for this preprint (which was not certified by peer review) is the author/funder, who has granted medRxiv a license to display the preprint in perpetuity.
It is made available under a CC-BY 4.0 International license .

Figure S2. Smooth average measurements of IgG, IgM, and IgA against SARS-CoV-2 spike protein receptor binding domain among PCR positive cases across time.

Limit of detection was artificially set at 0.3 µg/mL for IgM and IgG to match that of IgA. Points were jittered horizontally. A) All cases are shown. B) Cases are categorized by clinical severity.

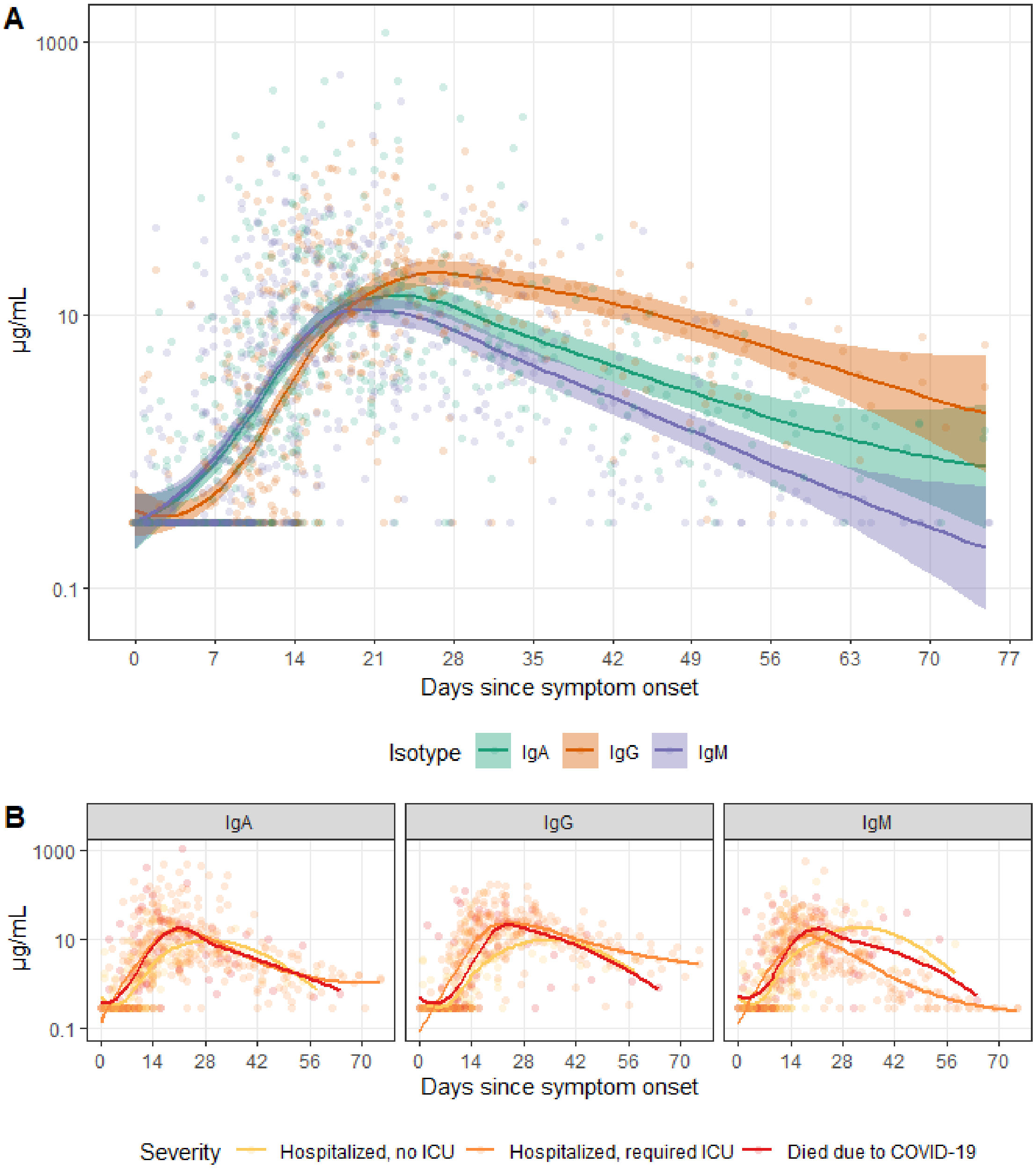

medRxiv preprint doi: https://doi.org/10.1101/2020.07.18.20155374.this version posted July 20, 2020. The copyright holder for this preprint (which was not certified by peer review) is the author/funder, who has granted medRxiv a license to display the preprint in perpetuity.
It is made available under a CC-BY 4.0 International license .

Figure S3. Individual trajectories for 16 randomly selected individuals with 4 or more measurements.

Patient ID numbers are shown in greys boxes.

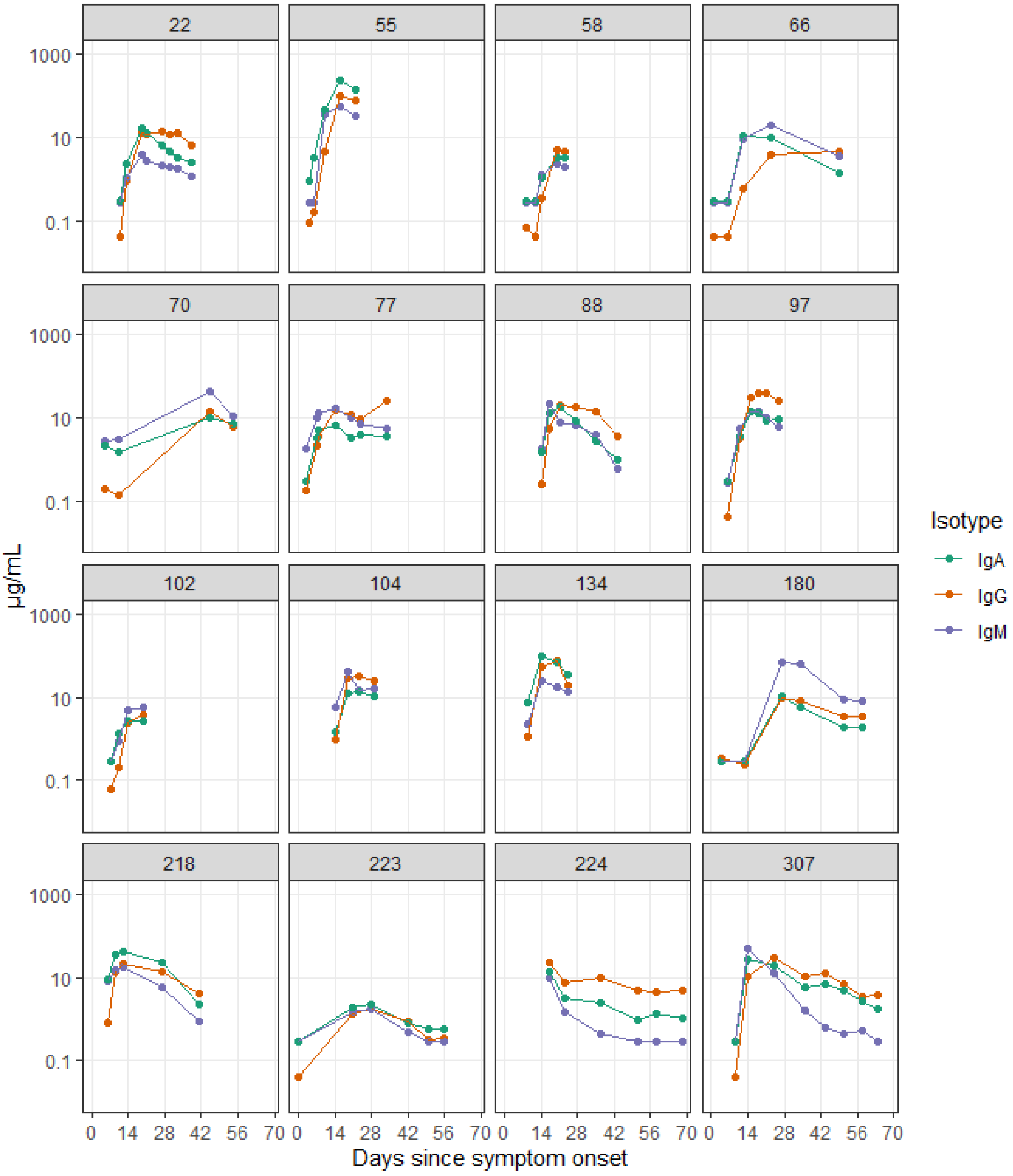

medRxiv preprint doi: https://doi.org/10.1101/2020.07.18.20155374.this version posted July 20, 2020. The copyright holder for this preprint (which was not certified by peer review) is the author/funder, who has granted medRxiv a license to display the preprint in perpetuity.
It is made available under a CC-BY 4.0 International license .

Table S1. Predictive accuracy of multiple isotypes for classifying controls and cases over time since symptom onset.

Random forest models were used to calculate cvAUC. The isotype cut-offs chosen for calculating sensitivity were the maximum concentration (μg/mL) found among pre-pandemic controls (IgG: 0.57, IgM: 2.63, IgA: 2.02). Samples with measurements above at least one cut-off were classified as cases.

Isotypes	Days since symptom onset	cvAUC (95% CI)	Sensitivity (95% CI)
IgA + IgG	≤7 days	0.64 (0.54-0.75)	0.12 (0.09-0.15)
	8-14 days	0.91 (0.86-0.96)	0.55 (0.51-0.59)
	15-28 days	0.99 (0.96-1.00)	0.97 (0.96-0.99)
	>28 days	1.00 (1.00-1.00)	0.97 (0.96-0.99)
IgM + IgG	≤7 days	0.64 (0.54-0.74)	0.10 (0.08-0.13)
	8-14 days	0.90 (0.85-0.96)	0.57 (0.53-0.61)
	15-28 days	1.00 (0.99-1.00)	0.98 (0.96-0.99)
	>28 days	0.98 (0.95-1.00)	0.97 (0.96-0.99)
IgM + IgA	≤7 days	0.61 (0.51-0.71)	0.11 (0.08-0.14)
	8-14 days	0.90 (0.84-0.96)	0.56 (0.51-0.60)
	15-28 days	0.99 (0.95-1.00)	0.95 (0.93-0.97)
	>28 days	0.98 (0.94-1.00)	0.69 (0.64-0.73)
IgM + IgA + IgG	≤7 days	0.64 (0.54-0.74)	0.12 (0.09-0.15)
	8-14 days	0.92 (0.86-0.97)	0.60 (0.56-0.64)
	15-28 days	1.00 (0.99-1.00)	0.98 (0.96-0.99)
	>28 days	1.00 (1.00-1.00)	0.97 (0.96-0.99)

medRxiv preprint doi: https://doi.org/10.1101/2020.07.18.20155374.this version posted July 20, 2020. The copyright holder for this preprint
(which was not certified by peer review) is the author/funder, who has granted medRxiv a license to display the preprint in perpetuity.
It is made available under a CC-BY 4.0 International license .

Figure S4. Measurements of IgG, IgM, and IgA against SARS-CoV-2 spike protein receptor binding domain among pre-pandemic controls and symptomatic PCR positive cases.

Black dashed line is at 0.57 µg/mL for IgG, 2.63 µg/mL for IgM, and 2.02 µg/mL for IgA.

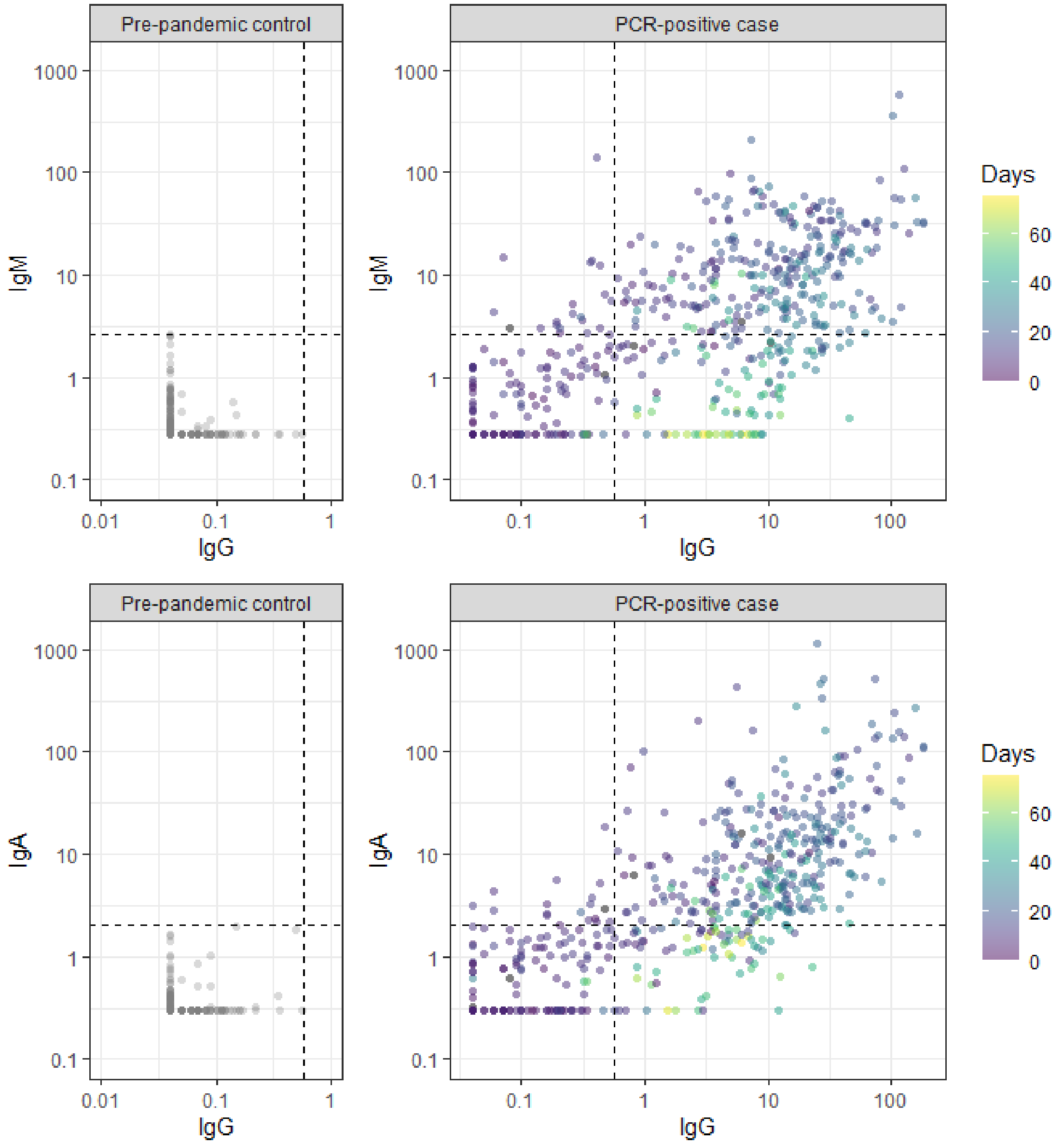

medRxiv preprint doi: https://doi.org/10.1101/2020.07.18.20155374.this version posted July 20, 2020. The copyright holder for this preprint (which was not certified by peer review) is the author/funder, who has granted medRxiv a license to display the preprint in perpetuity.
It is made available under a CC-BY 4.0 International license .

Figure S5. Receiver operating characteristic curve from random forest models and isotype contributions.

Each panel shows the ROC curves for cross-validated random forest models fit to serological measurements taken (A) under 7 days (cvAUC: 0.64), (B) 8-14 days (cvAUC: 0.92), (C) 15-28 days (cvAUC: 1.00) and over 28 days (cvAUC: 1.00) after symptom onset of PCR positive cases and pre-pandemic controls. Each blue line is one of ten cross-validated ROC curves for a specific time point. Median relative importance of each serological marker is shown in each bar graph.

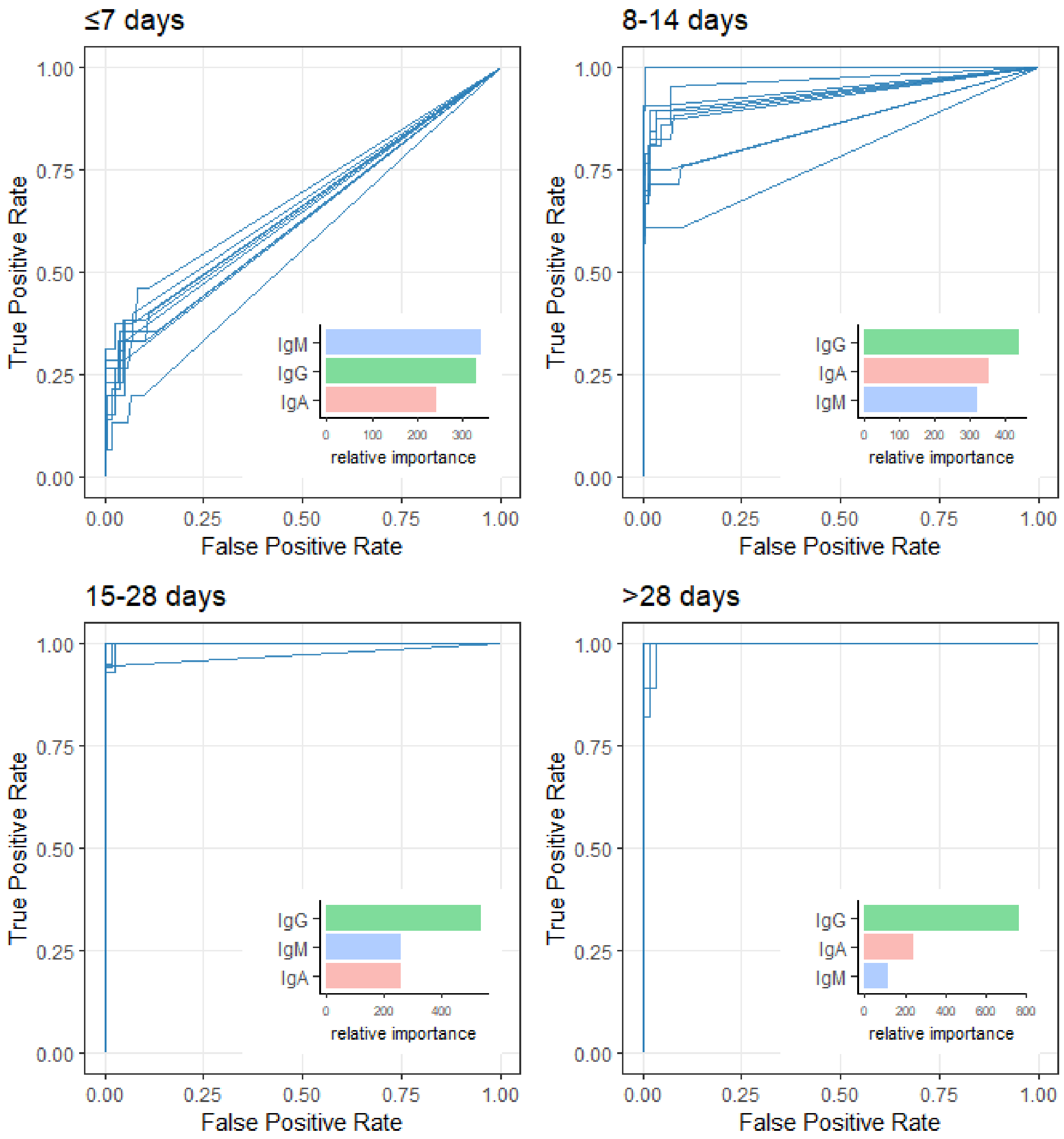

medRxiv preprint doi: https://doi.org/10.1101/2020.07.18.20155374.this version posted July 20, 2020. The copyright holder for this preprint (which was not certified by peer review) is the author/funder, who has granted medRxiv a license to display the preprint in perpetuity.
It is made available under a CC-BY 4.0 International license .

Table S2. Parametric estimates of median time to seroconversion for each isotype by different patient characteristics.

The isotype cut-offs chosen for seroconversion were the maximum concentration (μg/mL) found among pre-pandemic controls (IgG: 0.57, IgM: 2.63, IgA: 2.02). All models assumed that time to event followed a Weibull Distribution. Bootstrap 95% confidence intervals are shown in parentheses. Not Hospitalized and Hospitalized, no ICU were combined due to small sample size.

Isotype	Characteristic		50th percentile (95% CI)	Difference (95% CI)
IgA	Age			
		<65 years	11.9 (10.7-13.2)	
		≥65 years	13.3 (10.2-17.3)	1.4 (-1.5-5.4)
	Sex			
		Male	11.8 (10.6-13.1)	
		Female	11.5 (9.3-14.1)	-0.3 (-2.6-2.4)
	Severity			
		Not Hospitalized / Hospitalized, no ICU	12.2 (10.9-13.5)	
		Hospitalized, required ICU	9.3 (7.2-11.9)	-2.9 (-5.0--0.4)
		Died due to COVID-19	10.1 (7.2-14.1)	-2.1 (-5.0-1.6)
IgG	Age			
		<65 years	10.9 (10.0-11.9)	
		≥65 years	12.6 (10.4-15.2)	1.7 (-0.3-4.0)
	Sex			
		Male	10.9 (9.9-11.8)	
		Female	10.8 (9.2-12.6)	-0.1 (-1.7-1.5)
	Severity			
		Not Hospitalized / Hospitalized, no ICU	11.3 (10.3-12.3)	
		Hospitalized, required ICU	7.4 (6.2-8.8)	-3.8 (-5.2--2.5)
		Died due to COVID-19	11.6 (8.4-15.8)	0.4 (-2.7-4.1)
IgM	Age			
		<65 years	12.1 (10.8-13.7)	
		≥65 years	12.4 (9.5-16.0)	0.3 (-2.6-3.8)
	Sex			
		Male	12.2 (10.9-13.8)	
		Female	16.0 (11.4-22.5)	3.8 (-0.5-9.9)
	Severity			
		Not Hospitalized / Hospitalized, no ICU	12.3 (10.8-14.0)	
		Hospitalized, required ICU	10.1 (7.4-13.7)	-2.2 (-4.8-1.1)
		Died due to COVID-19	9.9 (6.4-14.4)	-2.5 (-5.9-2.0)

medRxiv preprint doi: https://doi.org/10.1101/2020.07.18.20155374.this version posted July 20, 2020. The copyright holder for this preprint (which was not certified by peer review) is the author/funder, who has granted medRxiv a license to display the preprint in perpetuity.
It is made available under a CC-BY 4.0 International license .

Figure S6. Measurements of IgG, IgA, and IgM against the RBD of other coronaviruses among pre-pandemic controls and PCR positive cases.

Each dot represents a unique measurement of a serological marker (Row A: IgG, Row B: IgA, Row C: IgM) in pre-pandemic controls (left panels) and PCR positive cases (right panels) for each coronavirus. Each line connects measurements (dots) for individuals.

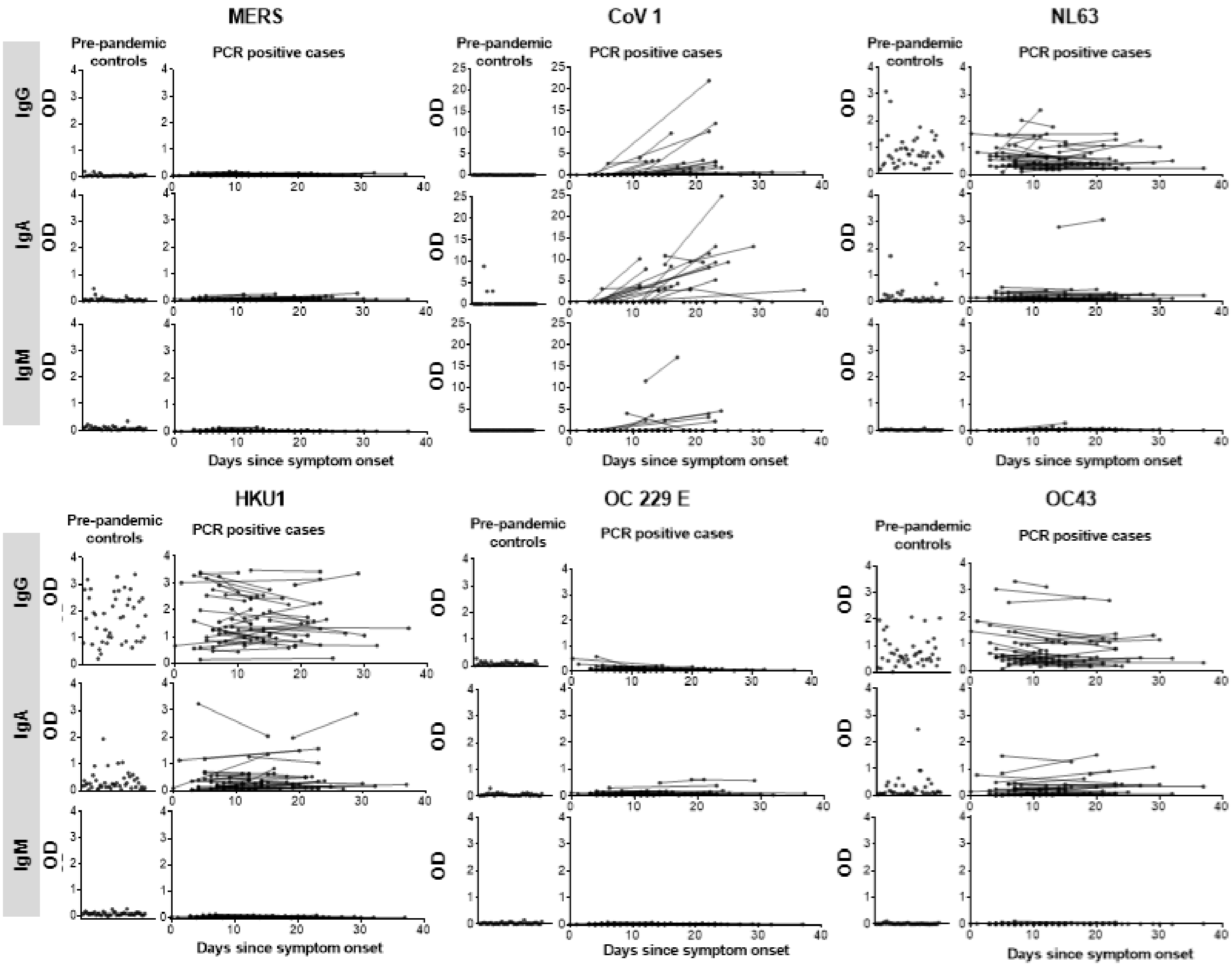

medRxiv preprint doi: https://doi.org/10.1101/2020.07.18.20155374.this version posted July 20, 2020. The copyright holder for this preprint (which was not certified by peer review) is the author/funder, who has granted medRxiv a license to display the preprint in perpetuity.
It is made available under a CC-BY 4.0 International license .

Figure S7. Correlation between plasma and dried blood spot measurements (DBS).

Plot of anti-RBD antibody IgG measurement in plasma versus DBS of 20 COVID cases (at 2 timepoints) and 20 pre-pandemic controls. The Pearson correlation coefficient (r) is shown. The dotted gray lines represent the concentration cut-off for seropositivity with plasma.

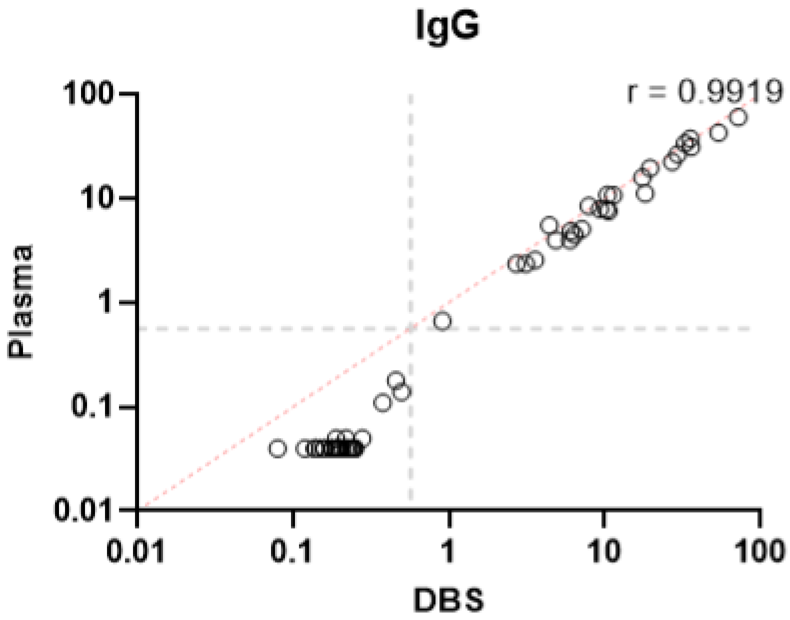

Supplement: 1 [file NIHPP2020.07.18.20155374-supplement-1.pdf]
